# Spontaneous Imbibition of Bicontinuous Microemulsions into Hydrophilic and Hydrophobic Controlled Pore Glasses

**DOI:** 10.1021/acsomega.6c00570

**Published:** 2026-05-20

**Authors:** Margarethe Dahl, Larissa Doll, Christian Tesch, Benjamin Paul, Jessica Hübner, Brigitte Tiersch, Thomas Hellweg, Stefan Wellert

**Affiliations:** † 26524 Technische Universität Berlin, Institut für Chemie, Straße des 17. Juni 135, Berlin 10623, Germany; ‡ Universität Potsdam, Institut für Chemie, Karl-Liebknecht-Straße 24-25, Potsdam 14476, Germany; § 9167 Universität Bielefeld, Institut für Chemie, Physikalische und Biophysikalische Chemie, Universitätsstraße 25, Bielefeld 33615, Germany

## Abstract

The effects of hard confinement are of great importance for many applications and also from a scientific point of view. These effects include a reduction in degrees of freedom and changes in phase behavior, structure, and molecular interactions. Simple and complex fluids can enter and occupy a porous matrix by imbibition. In this work, the results of an investigation into the spontaneous imbibition of bicontinuous microemulsions, serving as a model system for a complex fluid, into columns of granular, controlled pore glasses (CPG) are reported. The spontaneous imbibition of 1-octanol, a simple fluid with viscosity and surface tension similar to the bicontinuous microemulsion, is investigated for comparison. Controlled pore glasses with three different pore diameters between 11 and 112 nm with hydrophilic and hydrophobic surfaces are chosen. Two imbibition regimes were observed for the three investigated controlled porous glasses. Comparatively fast imbibition occurs due to the presence of voids between the individual CPG grains, while the uptake of liquids into the actual pores is much slower.

## Graphical abstract

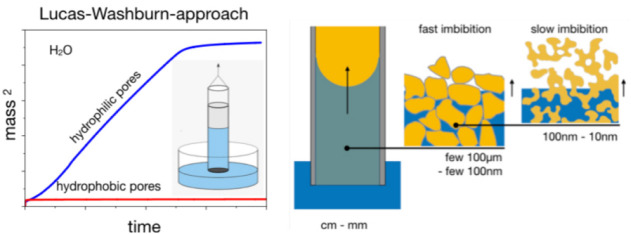

## Introduction

The term complex fluid is defined differently across various scientific disciplines. In this work, we adopt the definition used in the context of soft condensed matter physics and physical chemistry, as outlined in the literature.^1^ According to this definition, complex fluids are characterized by the presence of a mesoscopic length scale, which is intermediate between the molecular and macroscopic levels and plays a key role in determining their properties. In the systems discussed here, this mesoscopic scale includes, for example, the size of spherical or rod-like micelles (typically a few nanometers), the Flory radius or persistence length of flexible polymer chains (10–100 nm), the radius of microemulsion droplets or vesicles, the domain size of bicontinuous microemulsions, or the dimensions of colloidal particles (generally 10–100 nm). In addition, complex fluids often display unusual rheological behavior, such as shear thinning or thickening, stress-dependent viscoelasticity, and exceptional structural flexibility. These behaviors can be influenced even by weak forces, such as thermal fluctuations on the order of a few *k*_B_ *T*.

A microemulsion forms spontaneously and is a macroscopically homogeneous, thermodynamically stable mixture of at least three components: a hydrophilic, a hydrophobic, and an amphiphilic species.^2^ Typically, water acts as the hydrophilic component, oil as the hydrophobic one, and the surfactant positions itself at the interface, reducing the interfacial tension and stabilizing the microemulsion. Depending on composition, these systems can form oil-in-water (O/W), water-in-oil (W/O), or bicontinuous structures, in which continuous domains of both oil and water coexist. A bicontinuous microemulsion is a sponge-like structure formed by equal volumes of continuously connected and interwoven immiscible oil and water, stabilized by the minimal required amount of surfactant. Their equilibrium structure is readily tunable through composition and temperature. For these reasons, bicontinuous systems have frequently been employed as model systems for phenomena related to cytoplasmic crowding,^3,4^ membrane networks,^5,6^ and phase-separated biomolecular systems.^7^ In this sense, their relevance extends beyond soft matter science to areas including materials science and, in certain contexts, cell biology.

We used a bicontinuous microemulsion from the ternary phase system C_10_E_4_/H_2_O/*n*-octane as a model for a nanostructured, complex liquid. Notably, these microemulsions are capable of wetting both hydrophilic and hydrophobic planar surfaces.^8,9^ The near-surface structure in a bicontinuous microemulsion at planar surfaces was investigated with neutron reflectometry and small angle neutron scattering under grazing incidence.^10^ These various investigations revealed a layered structure in the vicinity of the surface which is followed by a transition region that merges into the well-known bulk structure.

Moreover, the interaction with a planar confinement also changes the thermal fluctuation spectrum of the surfactant membrane as was seen with neutron spin–echo spectroscopy.^11^ Given these unique properties, we are particularly interested in how such a soft, nanostructured liquid behaves when confined within rigid, nanoscale pores comparable in size to colloidal structures. In many applications, interactions between microemulsions and solid surfaces play a critical role. This is especially relevant in systems involving porous media, where the internal surface area such as that found in natural rocks is substantial.

Soft matter in hard confinement has also been extensively studied from a theoretical perspective. For example, the confinement of colloid–polymer mixtures between symmetric hard walls has been shown to exhibit capillary-condensation–like behavior,^12^ while phase separation of mixtures is predicted for asymmetric walls, where preferential localization of components can occur at the interfaces. Particular attention has been devoted to confinement effects on anisotropic colloids, such as rods, dumbbells, and ellipsoids.^13,14^ More recently, theoretical modeling has also incorporated active colloids under confinement. In addition, considerable work has focused on how confinement can actively steer the emergence or suppression of collective phenomena in space and time in various colloidal systems, which has significant implications for material design, biomedical applications, and the control of active matter.^15^

In addition to ordered mesoporous materials like MCM or SBA-type silica, so-called controlled pore glasses (CPGs) are widely used. CPGs are meso- or macroporous silica materials produced by leaching the borate phase from phase-separated alkali borosilicate glass. A well-known example is Vycor glass from Corning Glass Works. The resulting silica framework consists of a sponge-like network of interconnected pores, with diameters ranging from 7.5 to 3000 nm and specific surface areas between 40 and 300 m^2^/g. This distinguishes them from other silica adsorbents such as silica gel, which can achieve specific surface areas of up to 800 m^2^/g. CPGs possess narrow pore size distributions and higher chemical and mechanical resistances due to thicker silica walls.^16^ The pore structure of CPGs has been extensively characterized using techniques such as electron microscopy and small-angle scattering (SAS),^17–19^ mercury intrusion and gas sorption, and alternative methods like NMR cryo-porometry and thermo-porometry.^20,21^ Their well-defined pore network and large surface area make them ideal for studying processes such as imbibition and adsorption. As such, they are widely used in chromatography^22,23^ and for enzyme immobilization, which is relevant in biocatalysis, biosensor development, and controlled drug delivery.^24,25^

In the context of imbibition, porous materials are often idealized as vertical bundles of capillaries.^26,27^ Spontaneous imbibition, the capillarity driven rise of a liquid within pores, is a common phenomenon observed in everyday life (eg., paper towels absorbing spilled liquids).

A widely used method to quantify spontaneous imbibition is based on the Lucas–Washburn (LW) model.^26,28^ In this approach, imbibition is tracked either optically by observing the rising liquid front or gravimetrically by measuring mass uptake over time. Since the original work of Lucas and Washburn over a century ago, the LW model has been extended and refined to account for factors such as pore geometry, tortuosity, interfacial slip, and the presence of adsorbed layers.^29–31^

To date, most experimental studies of imbibition in porous materials have focused on simple fluids.^32–34^ Investigations involving complex fluids remain rare. For example, Dutta et al. studied the spontaneous imbibition of a binary toluene/*tert*-butanol mixture and observed phase separation during infiltration using neutron radiography.^35^ Gruener and Huber examined the imbibition of a liquid crystal into porous silica and found that shear-induced velocity gradients suppressed the formation of the smectic phase in favor of the nematic one.^36^

Thus, the experiments described in this paper aim to contribute to the understanding of complex fluid imbibition, particularly focusing on the fluid–solid interactions of a bicontinuous microemulsion with CPGs in powder form whose inner surfaces have been functionalized to be either hydrophilic or hydrophobic. The experimental scenario is sketched in Figure 1. On the left side the test fluid imbibes into the porous structure of the controlled pore glasses and the right image depicts the bicontinuous structure of the microemulsion. To provide a meaningful comparison, we also study the spontaneous imbibition of 1-octanol, a simple, molecular fluid with surface tension and viscosity values similar to those of the bicontinuous microemulsion. The experiments are carried out using columns of packed CPG grains with three different mean pore diameters ranging from 11 to 112 nm and with both, hydrophilic and hydrophobic surface modifications. This allows to study the imbibition process when the mean pore size of the CPG is smaller, very similar and larger than the mean distance of the oil and water domains inside the bicontinuous structure of the microemulsion.

**Figure 1:**
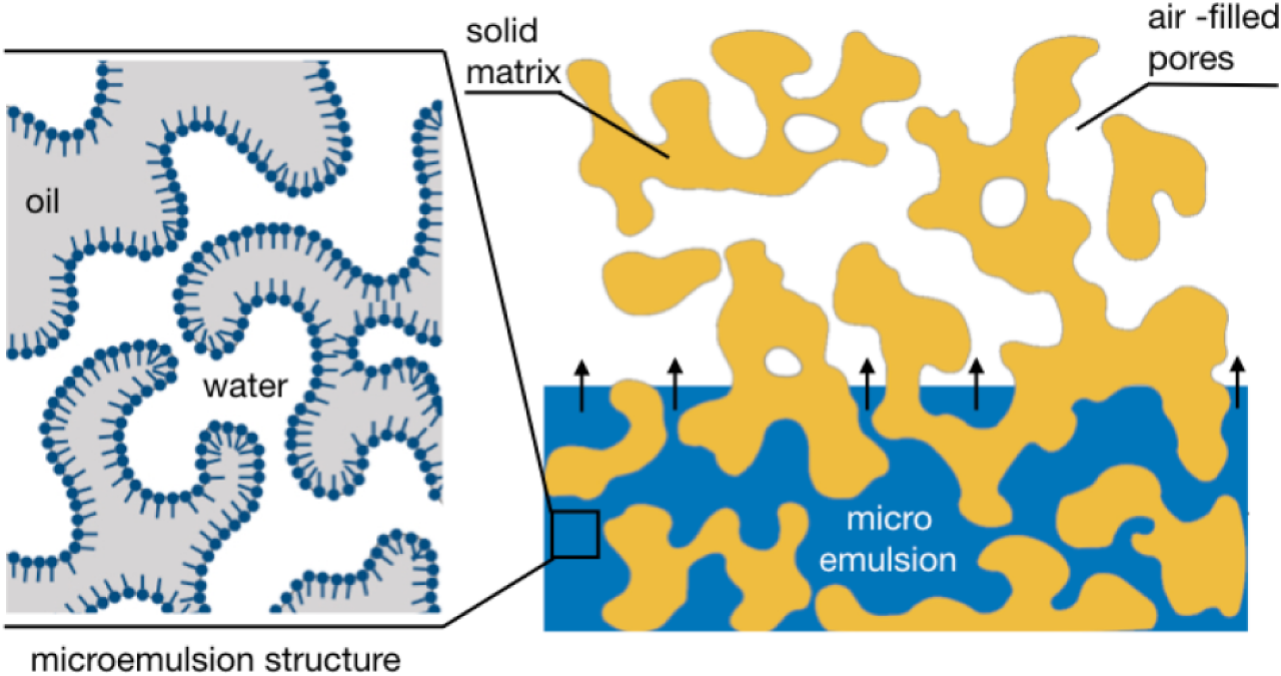
Schematic illustration of the experimental situation of spontaneous imbibition of a bicontinuous microemulsion into a porous sponge-like structure inside the CPG grains. A situation is sketched, when the pore size is larger than the size of the oil and water domains in the microemulsion. In both cases the important structural lengths range from 10 to a few 100 nm.

## Experimental Section

### Materials

Water was purified using a Milli-Q system (Millipore), reaching a final resistance of 18.2 MΩ. Tetraethylene glycol monodecyl ether (C_10_E_4_) (>95%) was purchased from Bachem (Bubendorf, Switzerland). *n*-Octane, dichlorodimethylsilane (DCDMS), 1-octanol and anhydrous toluene (99.8%) were purchased from Sigma-Aldrich. Sulfuric acid (95%) and an aqueous solution of hydrogen peroxide (30%, stabilized) were purchased from VWR Chemicals GmbH (Darmstadt, Germany). Chemicals were used as received.

#### Controlled Pore Glasses

CPG was used as porous powder material (pCPG). Their packing into columns is described afterward. The pCPGs were cleaned according to the following procedure. The pCPGs were treated with concentrated sulfuric acid for 48 h to remove any organic contamination. Then the powder was rinsed thoroughly with Milli-Q water and freeze-dried at −80 °C and 1 mbar. Until usage, the pCPGs were dried overnight in a vacuum furnace at 40 °C and stored in a desiccator. The surface polarity was altered following a modified procedure of Bosley and Clayton.^37^ Anhydrous toluene (2 mL) was transferred into a glass reactor, purged with nitrogen for 30 min, and stirred at 300 rpm. Afterward, 7 mL DCDMS was added to the toluene and stirred for 5 min at 600 rpm. The cleaned and dried pCPGs were placed separately in a PTFE tube. Afterward 2 mL of this solution was added to each PTFE tube and placed on an orbital shaker for 1 h at room temperature. The pCPGs were separated from the solution by centrifugation and washed twice with toluene, acetone, and Milli-Q water. The hydrophobically modified pCPGs are labeled as pCPG-CH_3_ and the hydrophilic pCPGs as pCPG-OH. To distinguish the pCPGs based on their pore size, this is included as a number in this designation. In the case of statements that are applicable to both pCPG-CH_3_ and pCPG-OH, the abbreviation pCPG is employed.

### Methods

#### Packing the CPG Particle Columns

To ensure reproducible imbibition measurements, it is essential to compact the particles in a uniform and robust manner. The details of the developed compaction procedure and its results are given in the SI, in particular in Figures S3 and S4.

#### cryoSEM

The structure of the bicontinuous microemulsion was captured using cryoSEM. A small droplet of the microemulsion was plunged into liquid nitrogen and freeze-fractured and etched at −98 °C for 45 s. The droplet was sputtered with platinum in a Gatan Alto 2500 cryo-preparation chamber. The images were recorded with a S-4800 instrument from Hitachi operated at an accelerating voltage of 2000 V; a magnification of 50,000 and 70,000 was achieved. The measurements were conducted at Universität Potsdam.

#### Microemulsion Sample Preparation

The simplest type of microemulsion is a ternary system based on a nonionic alkyl polyglycol ether (C_*i*_E_*j*_) surfactant. These microemulsions, e.g., the temperature-dependence of the phase behavior of the ternary system C_10_E_4_–H_2_O–*n*-octane, are well-characterized and documented in the literature.^38–41^ The index *i* gives the number of carbon atoms in the alkyl chain and *j* for the number of glycol ether units, the hydrophilic headgroup. The surfactant used in this work is tetraethylene glycol decyl ether C_10_E_4_. Ternary systems based on C_*i*_E_*j*_ surfactant exhibit all properties of more complex and technically relevant systems and are therefore the ideal model system.^2^ The phase behavior of the system depends on the composition and the temperature and can be depicted in a phase prism, shown in Figure 2. For an easier discussion, the common definitions are introduced.^42^ The volume fraction of water (ϕ_*w*_) can be expressed in terms of the water (*V*_water_) and oil volume (*V*_oil_) as ϕ_*w*_ = *V*_water_/(*V*_water_ + *V*_oil_). The surfactant concentration γ is usually expressed as a mass concentration using the surfactant mass *m*_surfactant_ as γ = *m*_surfactant_/(*m*_surfactant_ + *m*_water_ + *m*_oil_).

**Figure 2:**
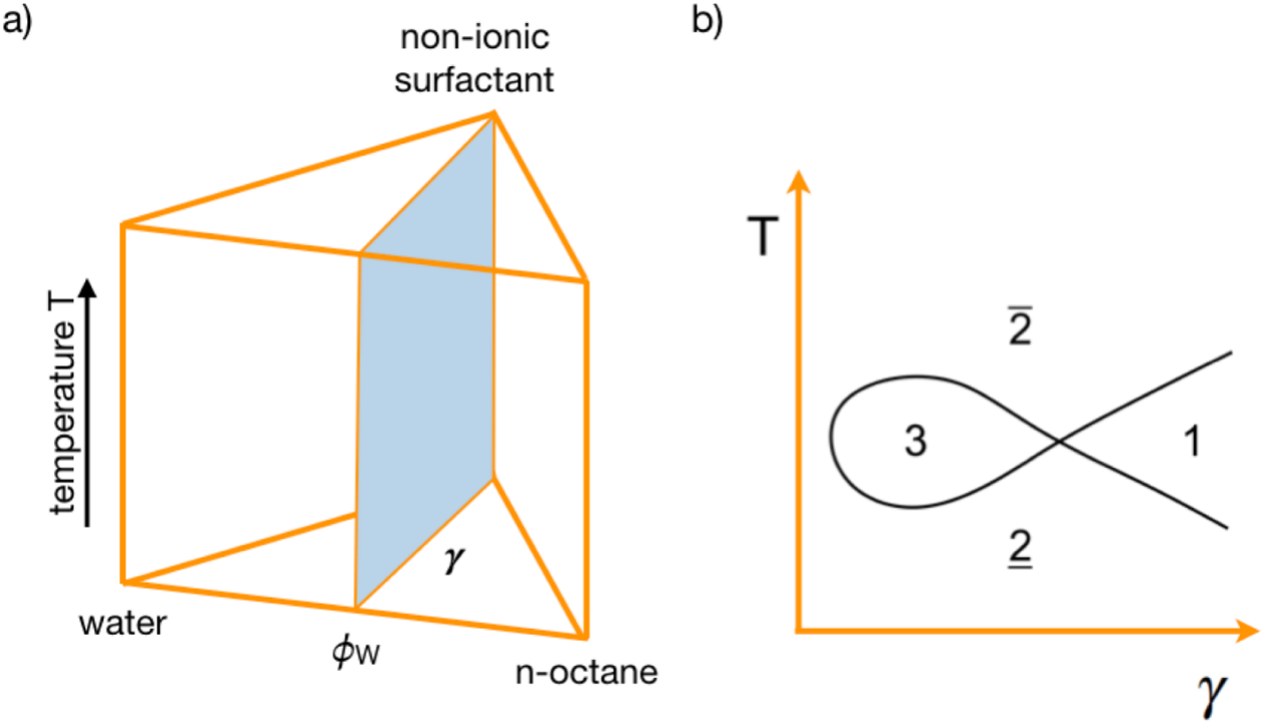
(a) Scheme of the three-dimensional phase prism based on (C*_i_* E*_j_*) surfactant as a function of the temperature T in the vertical direction. (b) Vertical cut (light blue area) through the phase prism at a fixed oil-to-water ratio ϕ*_w_* = 0.5, forming the two-dimensional fish-type diagram.

Vertically cutting the prism at the constant volume ratio of water (ϕ_*w*_ = 0.5) leads to the fish-shaped diagram (Figure 2), known as the Kahlweit fish.^38,39^ The numbers written in the phase diagram indicate the numbers of phases. The diagrams show two different two-phase regions, 2̅ denotes the coexistence of a surfactant-rich oil phase with an excess water phase, while the opposite case of a surfactant-rich water phase with an excess oil phase is denoted as 2̲. In the three-phase region a bicontinuous middle phase is formed after adding surfactant to the otherwise immiscible oil and water. An increase in the surfactant concentration increases the size of this middle phase. This can be found in the fish body in the diagram. The water and oil domains are separated by a monolayer of surfactant film and form a sponge-like network. At a characteristic surfactant concentration, the one-phase region is reached. It is the minimal amount of surfactant needed to disperse equal parts of oil and water homogeneously. Further addition of surfactant leads to lamellar structures after leaving the bicontinuous region.

The bicontinuous microemulsion was prepared using equal volumes of *n*-octane and heavy water, corresponding to a water volume fraction of ϕ_*w*_ = 0.5 and a surfactant concentration of γ = 0.1284. To ensure that the samples are bicontinuous and monophasic, the microemulsion samples were stored in a thermostated cabinet at T = 22.5 °C prior to all measurements.

Figure 3 shows cryo-SEM images of the investigated bicontinuous microemulsion which confirm the bicontinuous structure. Please note that due to the finite duration of the freezing process the structure was qualitatively confirmed. Additionally, the structural parameters were quantitatively characterized with small angle neutron scattering and compared to published data.^43^ The interdomain distance d_TS_ between the oil and water phases in the microemulsion was determined as d_TS_ = 33 nm. The correlation length of this bicontinuous structure was found to be ξ_*TS*_ = 22 nm. The expected structural parameters of the bicontinuous microemulsion as given in the literature were confirmed by those measurements.^44^ Compared with the mean pore diameter $d_{N_{2},\mathrm{Hg}}$ of the three investigated pCPGs, this allows to study the imbibition when $d_{N_{2},\mathrm{Hg}}<d_{TS}$ , $d_{N_{2},\mathrm{Hg}}\approx d_{TS}$ and $d_{N_{2},\mathrm{Hg}}>d_{TS}$ .

**Figure 3:**
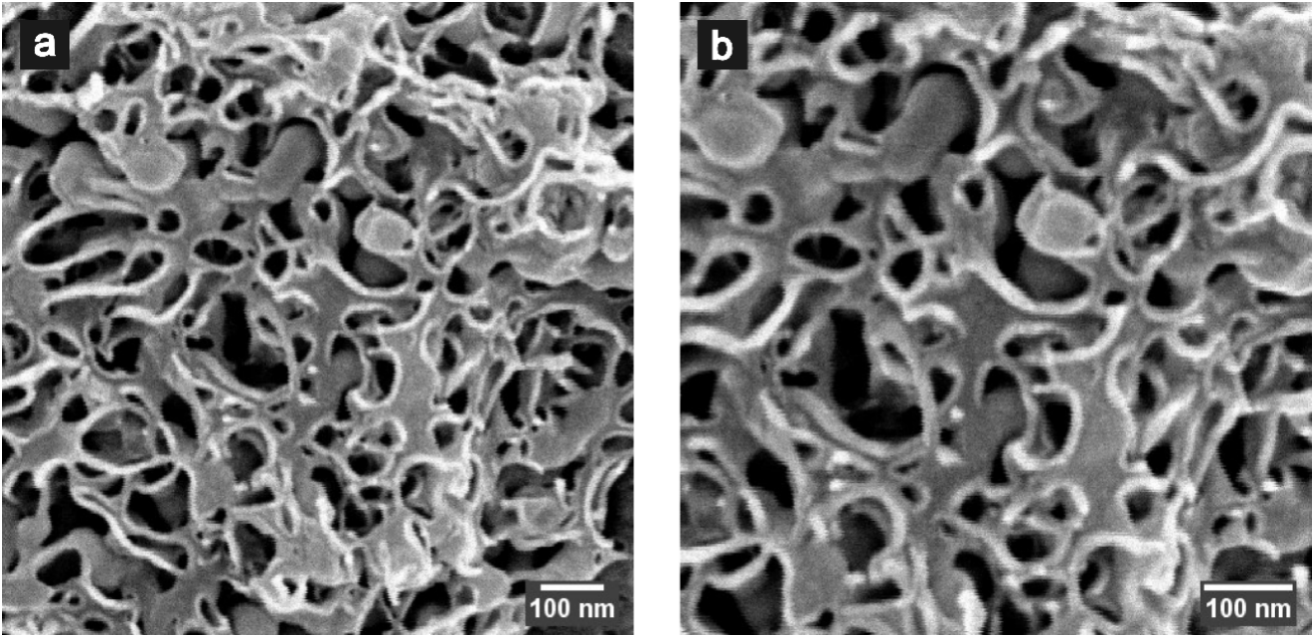
Cryo-SEM micrographs of the bicontinuous microemulsion in bulk at a magnification of 50,000 (a) and 70,000 (b).

#### Gravimetric Measurement of the Spontaneous Imbibition

Spontaneous imbibition is the rise of a liquid due to capillary forces and can be studied gravimetrically. The general setup for this method is illustrated in Figure 4. Glass tubes with an inner diameter of 8.5 mm and a height of 100 mm are used. The glass frit is covered with a filter paper to avoid clogging. The tube is filled with 0.5 g pCPG and compacted as described in the following section. Then the glass tube was placed into a tensiometer DCAT 11 from DataPhysics GmbH. The test liquid was filled into the reservoir below the glass tube. The sample tube was immersed automatically into the test liquid. The imbibition process starts immediately after the contact. The mass uptake was measured every 0.2 s. The measurement was terminated when the entire packed pCPG was filled with the liquid. The imbibition experiment of each liquid into every porous glass was repeated three to five times.

**Figure 4:**
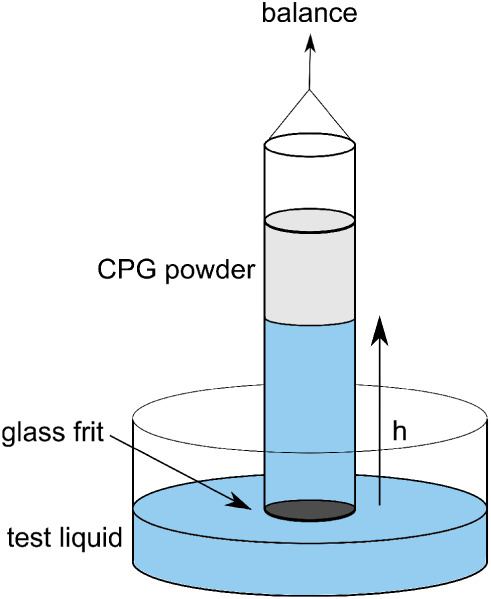
Scheme of experimental setup for studying the spontaneous imbibition. Glass tubes filled with a compacted CPG grains is brought into contact with a test liquid. The rise of the liquid is measured as a mass increase.

The capillary rise of a liquid into a porous medium can be described by the LW equation, which yields the height of the rising liquid depending on the square root of the time $\sqrt{t}$ with C_*h*_ as a proportionality factor which involves properties of the rising liquid and the pores:^26,28^

1 $$h\left(t\right)=C_{h}\sqrt{t}$$

If the rise of the liquid is followed gravimetrically, the rise is observed by a mass increase and the LW equation can be written as^45^

2 $$m\left(t\right)=C\sqrt{t}=\epsilon A\rho \sqrt{\frac{r_{p}\sigma_{l}\cos\theta }{2\tau \eta }t}$$

where C is the imbibition coefficient, defined by the porosity ϵ, the cross-sectional area of the sample *A* and the pore radius *r*_*p*_. The properties of the rising liquid are expressed by ρ, σ_*l*_ and η for the density, surface tension and dynamic viscosity, respectively. When plotted, it is common to use m^2^(t) = C^2^t to show the data in a linear form.

This general equation simplifies the porous medium to a bunch of identical parallel capillaries without any interconnections. Thus, the tortuosity τ, which is widely used as a critical parameter to measure the sinuosity of a porous material and predict transport properties of it, is neglected. For the sponge-like network of the CPG, a higher tortuosity τ = 3.6 has been reported.^34^

To determine the advancing contact angle of porous materials with the LW approach, imbibition experiments are first conducted with a reference liquid that completely wets the surface (θ = 0). From the slope of the linear regime, the proportionality constant C is obtained, assuming the $\sqrt{t}$ dependency holds.^27^ In this work, water and *n*-octane are among the main components of the bicontinuous microemulsion under study. Therefore, they were disregarded as reference liquids. The selection of the reference liquid is critical, as it can lead to arbitrary apparent contact angles and, consequently, affect the reliability of the method.^46^ Another limitation of determining contact angles via the LW approach is that the maximum measurable angle is θ = 90° (see eq 1), which is lower than the advancing water contact angle θ_*a*_ measured on the hydrophobic planar model surface Table 1.

**Table 1:** Characteristics of the Powder CPG (pCPG)^a^

Designation	$d_{N_{2},\mathrm{Hg}\;}\left(\mathrm{nm}\right)$	*V*_*P*_ (mL/g)	ϵ	*A*_*S*_ (m^2^/g)
pCPG11	11.4	0.67	0.60	201.0
pCPG50	46.0	1.16	0.72	68.1
pCPG100	112.0	1.57	0.78	35.9

a Pore diameter $d_{N_{2},\mathrm{Hg}}$ , pore volume *V_P_*, porosity ϵ, which is the fraction of pore volume to total volume, and the specific surface area *A_s_* obtained from Hg-intrusion and N_2_-sorption measurements.

## Results and Discussion

### Wetting Properties at Planar Model Surfaces

The equilibrium contact angle provides a measure of a liquid’s wettability on a solid surface. Representative examples of water contact angles on hydrophilic and hydrophobic surfaces are shown in Figure 5a_1_ and a_2_. On the hydrophilic Si–OH surface, the water droplet spreads almost completely, resulting in a contact angle below the detection limit of 10° and therefore barely visible (Figure 5a_1_). In contrast, on the hydrophobic Si–CH_3_ surface, the contact angle exceeds 90°, as shown in Figure 5a_2_.

**Figure 5:**
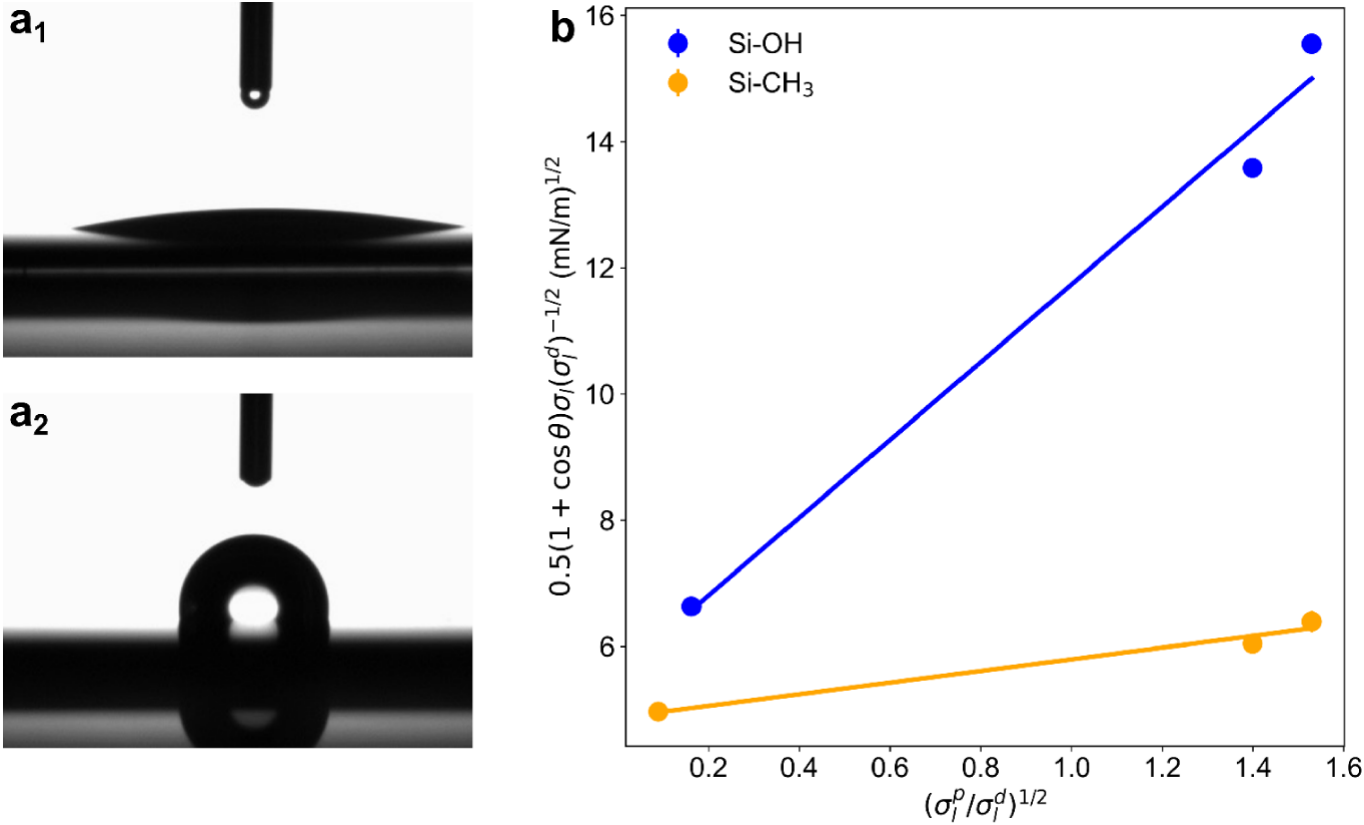
Static contact angle of water on hydrophilic (a_1_) and hydrophobic surface (a_2_). Plot for the determination of the surface free energy for hydrophilic and hydrophobic model surfaces (b). Further details of the measurements are given in Table S1 in the SI. The plot shows the linearized OWRK equation (eq S3 in the SI) combining contact angle θ and the polar and dispersive components of the liquids surface tension $\sigma_{l}^{p}$ and $\sigma_{l}^{d}$ . Slope and intercept in the plot yield the polar and the dispersive components of the solid substrate’s surface tension.

By measuring the contact angles of various probe liquids, the surface free energy can be determined using the Owens–Wendt–Rabel–Kaelble (OWRK) method.^47–49^ In this work, the OWRK method was used to determine the polar $\left(\sigma_{s}^{p}\right)$ and dispersive $\left(\sigma_{s}^{d}\right)$ components of the surface free energy for the model planar surfaces. Further details and results are presented in Figure 5b and summarized in Table S2 in the SI. The data show a significant reduction in surface free energy following hydrophobic modification, with a particularly strong decrease in the polar component and a slightly reduced dispersive component. The measured values are consistent with those reported in the literature.^50–53^ Since the hydrophobic functionalization of pCPG was carried out using the same procedure, a comparable reduction in surface energy is assumed for pCPG-CH_3_.

Following the surface energy analysis, the wettability of the bicontinuous microemulsion and its individual components was examined on the two model surfaces. Additionally, an aqueous C_10_E_4_ solution at a concentration of 0.5 mmol/L was tested. This concentration was selected because it lies just below the critical micelle concentration (CMC) of 0.66 mmol/L. The surface tension σ_*l*_, the density ρ, and the dynamic viscosity η of the tested liquids are summarized in Table 2.

**Table 2:** Surface Tension σ_l_, Density ρ and Dynamic Viscosity η of the Microemulsion (ME), Its Components and 1-Octanol at 23 °C

Liquid	σ_*l*_ (mN/m)	ρ (g/mL)	η (mPas)
Water	71.2	1.00	0.93
*n*-Octane	20.8	0.70	0.52
C_10_E_4 aq._	30.8	1.00	0.89
ME	21.6	0.87	6.25
1-Octanol	25.2	0.80	7.00

These measurements were conducted not only to characterize wetting behavior but also to assess the potential for preferential adsorption at the solid–liquid interface. To gain further insight into wetting dynamics, both advancing contact angles θ_*a*_ and receding contact angles θ_*r*_ were measured. The difference between these values, known as contact angle hysteresis, reflects the presence of metastable states at the contact line, often caused by surface heterogeneities.^54^ Contact angle hysteresis can also be interpreted as an activation energy barrier for changes in interfacial area.^55^ For *n*-octane, the contact angle could not be measured, as the liquid spread completely across both surfaces. As a result, no values are reported for *n*-octane in Table 3. A similar complete spreading behavior was observed for the bicontinuous microemulsion on the hydrophilic Si–OH surface.

**Table 3:** Advancing Contact Angle θ_a_, Receding Contact Angle θ_r_ and Contact Angle Hysteresis θ_hys_ for the Microemulsion and Its Components on Hydrophilic Si–OH and Hydrophobic Si–CH_3_ Surfaces^a^

	Water			C_10_E_4 aq._			ME		
Surface	θ_*a*_ (°)	θ_*r*_ (°)	θ_hys_ (°)	θ_*a*_ (°)	θ_*r*_ (°)	θ_hys_ (°)	θ_*a*_ (°)	θ_*r*_ (°)	θ_hys_ (°)
Si–OH	15 ± 1	7 ± 4	8 ± 5	13 ± 2	8 ± 3	5 ± 5	≈0	≈0	
Si–CH_3_	94 ± 2	84 ± 2	10 ± 4	48 ± 6	32 ± 1	16 ± 7	22 ± 3	11 ± 4	11 ± 7

a Contact angles below 20° should be interpreted with caution, as measurements in this range are known to be less reliable due to challenges in detecting the droplet profile accurately.^56^

The dynamic contact angle measurements demonstrate that the planar hydrophilic surface (Si–OH) is wetted by all investigated liquids. Due to the high surface energy of the Si–OH surface, which promotes spreading of the liquid in order to minimize the solid–air interfacial energy. *n*-Octane spreads completely on both surface polarities and exhibits the lowest contact angles among all tested liquids, consistent with its low surface tension. Water is the only liquid that does not wet the hydrophobic Si–CH_3_ surface. Interestingly, the aqueous C_10_E_4_ solution does wet this surface, likely due to the significant reduction in its surface tension, as shown in Table 2. A similar trend was observed in previous work.^53^ Early studies on liquid spreading on solid surfaces established that all liquids tend to wet high-energy surfaces, while wetting on low-energy surfaces depends primarily on the liquid’s surface tension.^57,58^ Specifically, liquids with low surface tension spread on low-energy surfaces, whereas those with high surface tension, such as water, do not. This relationship is illustrated in Figure 6, where the dynamic contact angle is plotted as a function of surface tension for the hydrophilic (Si–OH) and the hydrophobic (Si–CH_3_) surfaces.

**Figure 6:**
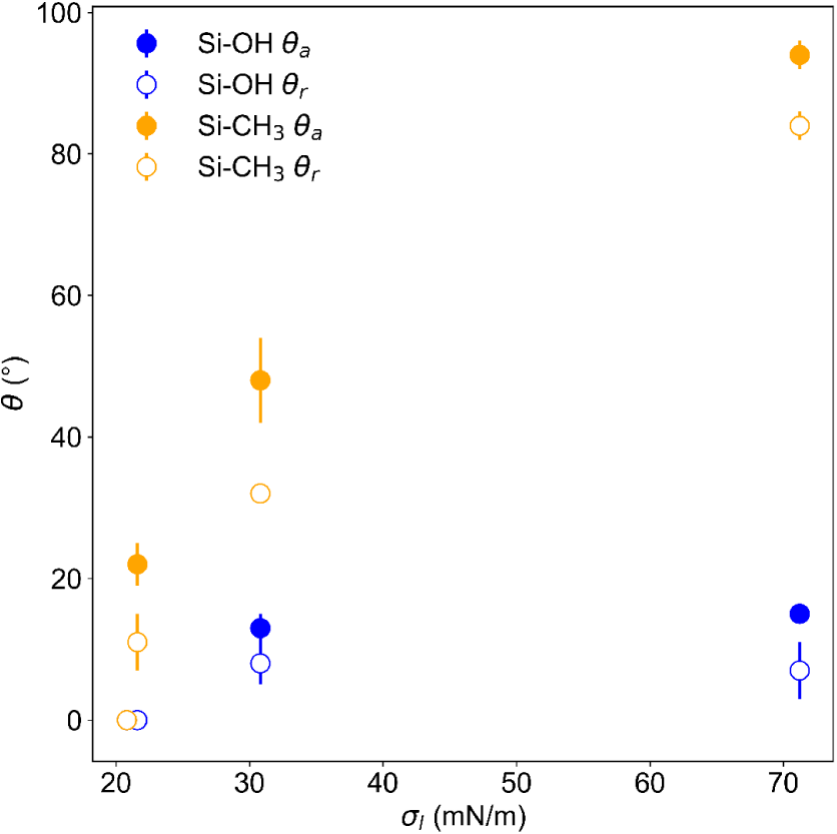
Advancing contact angle θ*_a_* (filled symbols) and receding contact angles θ*_r_* (open symbols) on hydrophilic (blue) and hydrophobic (orange) surfaces in dependence of the surface tension of the test liquid σ*_l_*.

The bicontinuous microemulsion (ME) wets both surfaces. On the hydrophilic surface, almost complete wetting occurs and the remaining contact angle is below the detection limit of 10 °C. On the hydrophobic surface, contact angles of θ_*a*_ = (22 ± 3)° and θ_*r*_ = (11 ± 4)° were measured. In general, the contact angle hysteresis on the hydrophobic surface is higher compared to Si–OH. This indicates a rougher surface which is caused by the chemical modification of the surface.^9^

### Spontaneous Imbibition into Columns of CPG Grains

#### Spontaneous Imbibition of Water into pCPGs

Before examining the spontaneous imbibition of the bicontinuous microemulsion, the rise of water into dry, air-filled columns of pCPG grains was investigated. As shown in Figure 7, images (a) and (b), beside the bicontinuous structure of the material, SEM images of the pCPG50-OH particles highlight their heterogeneity in size and shape, emphasizing the importance of achieving uniform and reproducible packing (Additional SEM images are shown in Figures S1 and S2 in the SI). To ensure consistent and thorough compaction across all experiments, 150 tapping cycles were applied to each filled tube which was found to be sufficient to achieve compacting the grains. The details of the filling and compacting procedure are given in the SI in Figures S3 and S4.

**Figure 7:**
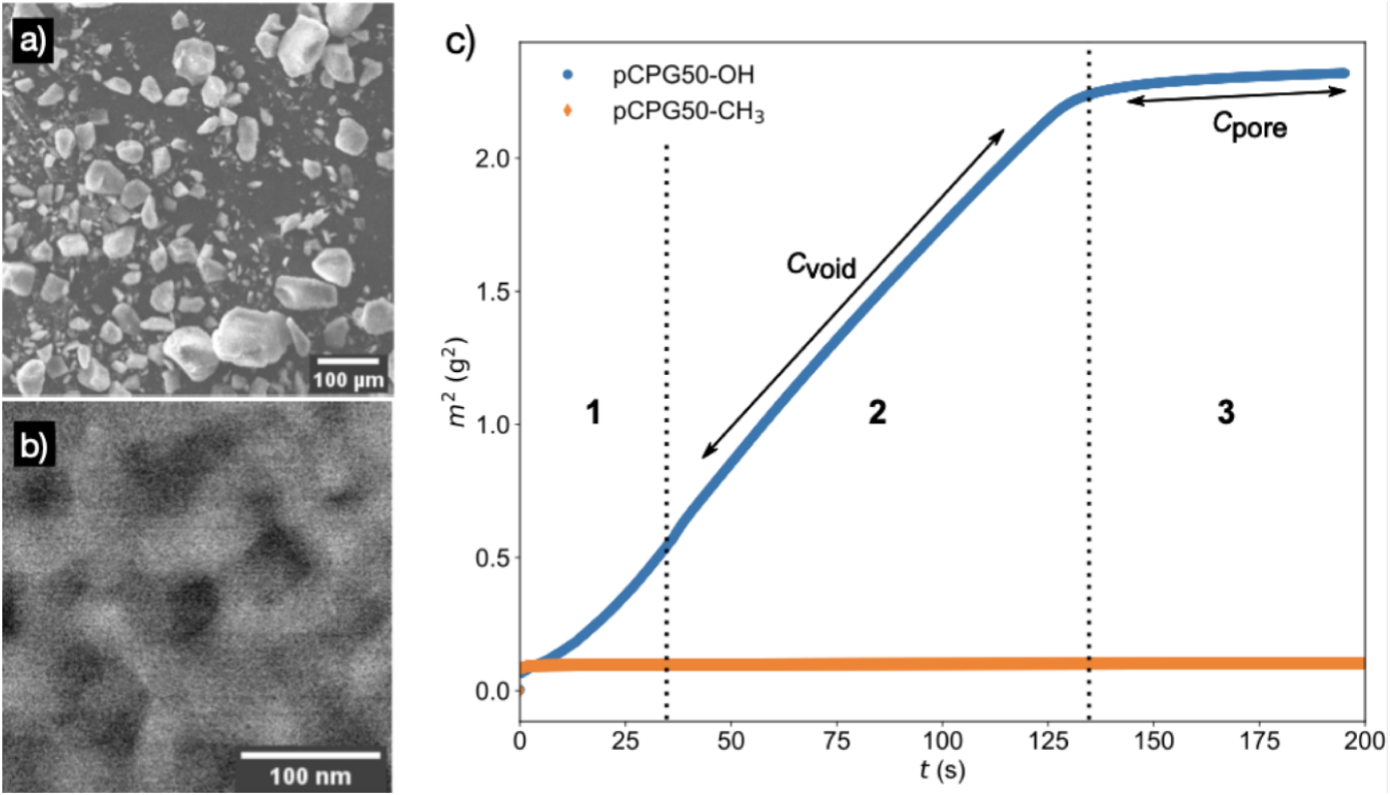
SEM micrographs of pCPG50-OH at magnifications of (a) 50 and (b) 50k. The white areas indicate the silicate skeleton of the porous material. c) Gravimetrical determination of the spontaneous imbibition of water into densely packed pCPG50-OH (blue) and hydrophobic (red) pCPG50-CH_3_ with a pore diameter of (48.0 ± 2.5) nm. Note that squaring the mass m gives a linear time t in the plots. Region 1 is the initial phase of the imbibition, the intergrain voids become filled in range 2 while the pores are filled in range 3. C*_void_* and C*_pore_* were determined from linear fits in ranges 2 and 3.

During the imbibition process, the invading water displaces the air within the porous network. An exemplary result of such a measurement is shown in Figure 7c, illustrating the spontaneous imbibition of water into both hydrophilic and hydrophobic pCPG50 samples with a nominal pore diameter of (48.0 ± 2.5) nm. The measured values of m­(t) are plotted as m^2^(t) = Ct which gives a linear form of the square of the mass vs a linear time t with a so-called Lucas–Washburn coefficient as proportionality factor. The imbibition behavior of water differs markedly between the two surface chemistries investigated here. A pronounced increase in mass uptake is observed for the hydrophilic pCPG-OH, whereas no measurable mass change occurs for the hydrophobically modified pCPG-CH_3_. This absence of imbibition indicates that water does not wet the pCPG-CH_3_, confirming the successful hydrophobic modification of the material.

In the case of the hydrophilic pCPG, the evolution of m^2^(t) exhibits three distinct regimes, as previously described in the literature.^27^ First, the three areas were visually identified and classified as ranges 1, 2, and 3. The classification is shown in Figure 7c using vertical dashed lines as an example. Ranges 2 and 3 were used for further analysis. The experimentally determined Lucas–Washburn coefficients C_*void*_ and C_*pore*_ for the second and third range were obtained by fitting the slopes of these respective regions and taking the square root. The resulting values of C_*void*_ and C_*pore*_ are listed in Table 4. The values C_*pore*_ and C_*void*_ were averaged for three to five imbibition runs from which the error was taken as the maximal deviation from the mean value.

**Table 4:** LW Coefficients, Experimentally Determined for the Imbibition into the Inter Particle Voids C_*void*_, the pCPG Pores C_*pore*_ and C_*LW*_ the Estimated Values According to Eq 2 for Imbibition into the Pores

pCPG	Rising liquid	C_*void*_ (mg s^–1/2^)	C_*pore*_ (mg s^–1/2^)	C_*LW*_ (mg s^–1/2^)
pCPG11-OH	H_2_O	99.5 ± 0.5	12.7 ± 0.2	7.5
	C_10_E_4 aq._	33.2 ± 2.4	26.8 ± 1.6	5.2
	*n*-Octane	55.2 ± 1.3	6.3 ± 0.4	3.9
	ME	23.7 ± 1.6	6.4 ± 0.8	1.4
	1-Octanol	20.3 ± 0.6	8.3 ± 0.3	1.3
pCPG11-CH_3_	H_2_O	0.0 ± 0.0	0.0 ± 0.0	0.0
	C_10_E_4 aq._	0.0 ± 0.0	0.0 ± 0.0	4.5
	*n*-Octane	76.9 ± 5.9	15.1 ± 1.2	4.0
	ME	9.9 ± 0.3	4.0 ± 0.4	1.4
	1-Octanol	33.8 ± 4.4	4.0 ± 0.3	1.3
pCPG50-OH	H_2_O	131.1 ± 4.6	25.0 ± 3.0	18.5
	C_10_E_4 aq._	110.5 ± 6.3	22.0 ± 2.0	12.8
	*n*-Octane	83.1 ± 2.0	12.7 ± 1.8	9.7
	ME	26.5 ± 0.6	9.5 ± 0.5	3.3
	1-Octanol	23.8 ± 2.0	3.7 ± 0.7	3.5
pCPG50-CH_3_	H_2_O	0.0 ± 0.0	0.0 ± 0.0	0.0
	C_10_E_4 aq._	0.0 ± 0.0	0.0 ± 0.0	11.1
	*n*-Octane	58.6 ± 2.8	14.2 ± 1.2	9.7
	ME	20.6 ± 2.9	3.7 ± 0.4	3.5
	1-Octanol	20.3 ± 0.6	3.5 ± 0.3	3.3
pCPG100-OH	H_2_O	162.8 ± 12.0	28.7 ± 2.7	30.8
	C_10_E_4 aq._	52.4 ± 2.5	19.0 ± 2.0	21.2
	*n*-Octane	69.6 ± 3.8	10.8 ± 0.7	16.1
	ME	19.7 ± 1.5	6.6 ± 1.0	5.5
	1-Octanol	26.1 ± 0.4	5.1 ± 0.7	5.9
pCPG100-CH_3_	H_2_O	0.0 ± 0.0	0.0 ± 0.0	0.0
	C_10_E_4__aq._	0.0 ± 0.0	0.0 ± 0.0	20.5
	*n*-Octane	55.6 ± 6.0	7.1 ± 0.6	16.1
	ME	18.8 ± 0.4	7.9 ± 0.6	5.4
	1-Octanol	14.7 ± 0.4	6.6 ± 0.8	5.8

Initially, the test liquid must penetrate and pass through the glass frit and filter paper before imbibition into the actual sample begins. In this first regime, inertial forces dominate, leading to a linear increase in m^2^(t). This section is prone to experimental errors and hence, is not further analyzed. The second regime is governed by capillary forces and at the end this regime, the pCPG-OH column becomes fully saturated with water, a state that can be confirmed visually.

In the third regime, the mass increase can reach a plateau when C_*pore*_ ≈ 0, indicating that the sample is either completely saturated with the test liquid or that gravitational forces have balanced the driving capillary pressure or. In materials with nanometer-scale pore radii, the Laplace pressure greatly exceeds the hydrostatic pressure, allowing the latter to be neglected.^34^ The extracted values of C_*void*_ and C_*pore*_ were compared to a theoretical parameter C_*LW*_ that was calculated according to the prefactor given in eq 2. For the calculation of C_*LW*_ the contact angle values for the respective test liquids were taken from the measurements on planar surfaces, pore radii were taken from the porosimetry measurements (given in Table 1) and a tortuosity of 3.6 as previously explained in the text was used.

#### Spontaneous Imbibition of Other Simple Liquids and a Bicontinuous Microemulsion into pCPGs

In addition to water, the spontaneous imbibition of *n*-octane, an aqueous C_10_E_4_ solution, the bicontinuous microemulsion, and 1-octanol was investigated. The physical properties of these liquids are summarized in Table 2. At the specified concentration, the bicontinuous microemulsion exhibits Newtonian fluid behavior, consistent with the other fluids studied.^59^ Consequently, comparison with 1-octanol helps to assess whether the structural complexity of the bicontinuous microemulsion affects its imbibition behavior.

Representative imbibition curves for pCPG50 are presented in Figure 8, for the case where $d_{N_{2},\mathrm{Hg}}\leq d_{TS}$ . The imbibition data pCPG11 and pCPG100, when $d_{N_{2},\mathrm{Hg}}<d_{TS}$ and $d_{N_{2},\mathrm{Hg}}>d_{TS}$ are provided in the SI in Figures S5 and S6. Except for the cases of water and the aqueous surfactant solution imbibing into pCPG-CH_3_, all curves display the three characteristic regions in m^2^(t) previously discussed in relation to Figure 7c. Among all tested liquids, water exhibits the steepest curve, corresponding to the largest slope in the second regime, which can be attributed to its high surface tension and low viscosity.

**Figure 8:**
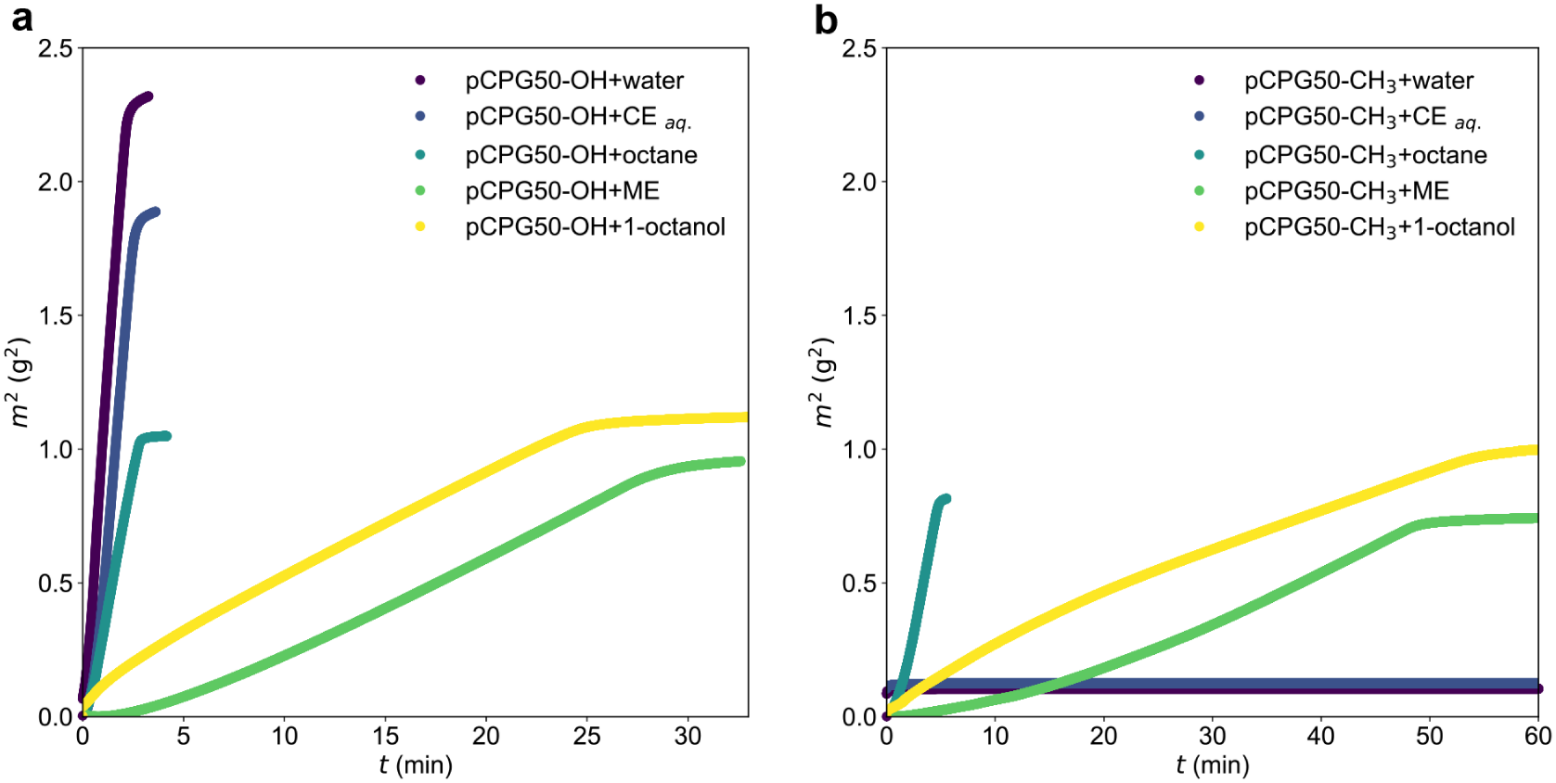
Imbibition into hydrophilic pCPG-OH (a) and hydrophobic pCPG-CH_3_ (b). Test fluids used in these measurements were water, aqueous C_10_E_4_-solution (CE_aq._), octane, the bicontinuous microemulsion (ME) and 1-octanol.

##### Imbibition of Aqueous Surfactant Solutions

The aqueous surfactant solution also imbibes rapidly into pCPG-OH but does not penetrate pCPG-CH_3_. This behavior is noteworthy, as the hydrophobic planar surface was found to be partially wetted by the same surfactant solution. A possible explanation is the reduction in surface tension, which lowers the Laplace pressure. However, a more plausible interpretation is the energetically unfavorable interaction between the aqueous phase and the hydrophobic pore surface.

The capillary rise of C_*i*_E_*j*_ solutions with varying concentrations in hydrophobic capillaries has been investigated. The rise is strongly dependent on the surfactant concentration. At low concentrations (0.5 mmol/L), the rise is much less pronounced and proceeds more slowly. The initial stage of the rise is limited by surfactant adsorption at the solid–liquid interface.^60^ At this concentration, the amount of surfactant present in the solution is insufficient to fully cover the internal surface of the pCPG. To coat the entire surface area of the pCPG50 column, approximately 6.3 × 10^19^ surfactant molecules would be required, assuming an area per molecule of 54 Å^2^.^44^ This corresponds to roughly 200 mL of the test solution, far exceeding the actual imbibed liquid volume of only 1–2 mL. Consequently, the situation cannot be directly compared to that of a planar surface due to the vastly larger surface area of the porous structure.

##### Imbibition of *n*-Octane

The imbibition of *n*-octane appears to be unaffected by the surface chemistry of the pCPG. In contrast, the imbibition of the bicontinuous microemulsion proceeds at a significantly slower rate compared to its pure components.

##### Imbibition of 1-Octanol

To determine whether this reduced rate is related to the intrinsic structural complexity of the microemulsion or to its macroscopic properties, such as viscosity, a comparative experiment was conducted using 1-octanol. This simple liquid exhibits viscosity and surface tension values comparable to those of the bicontinuous microemulsion. The resulting imbibition curves of 1-octanol closely resemble those obtained for the microemulsion.

##### Imbibition of a Bicontinuous Microemulsion

The imbibition curve of the bicontinuous microemulsion does not display any qualitative deviations from those of the simple liquids. Therefore, it is hypothesized that the slow imbibition of the bicontinuous microemulsion arises solely from its macroscopic physical properties, rather than from surfactant adsorption or diffusion processes. It should be noted that the gravimetric method employed here may not be ideally suited for detecting a potential phase separation. Previous gravimetric studies of the spontaneous imbibition of tert-butanol and toluene also showed no indication of separation; however, neutron radiography revealed the presence of two distinct advancing fronts, with the leading front corresponding to toluene.^35^

#### Discussion of the Observed Imbibition Mechanism

The packing of the irregularly shaped pCPG particles creates two distinct types of porosity: one at the micrometer scale, corresponding to the voids between the irregularly shaped particles due to their imperfect packing, and another at the nanometer scale, representing the internal porosity of the individual pCPG particles, as illustrated in Figure 9.

**Figure 9:**
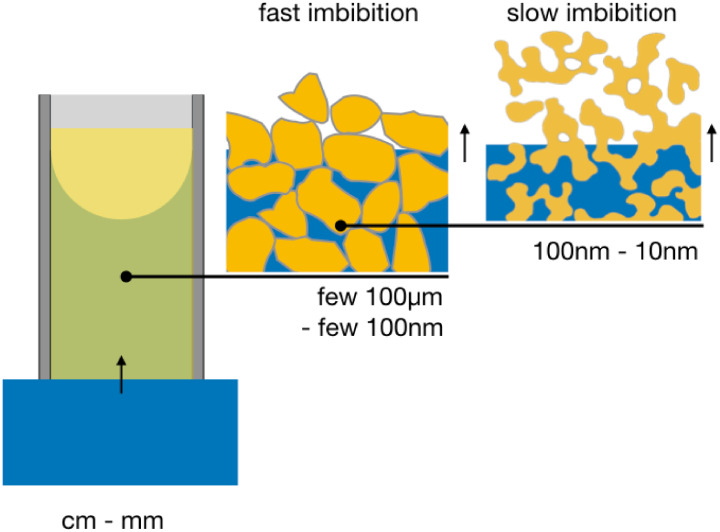
Schematics of the different types of spontaneous imbibition into pCPG. The macroscopically observed rise of the imbibition front is a combination of slow imbibition into the pores in the CPG grains and the filling of the spaces between the individual, irregularly shaped grains.

The mass increase associated with the rising liquid is directly proportional to the pore size $d_{N_{2},\mathrm{Hg}}$ given in Table 1, leading to two distinct slopes in the imbibition curve for the respective porous structures. Because the interparticle voids are larger and more irregularly shaped than the nanopores within the pCPG particles, the mass uptake in these voids is expected to be both greater and faster than that within the internal pore network. A similar observation was previously reported for the imbibition into packed porous particles.^61^ In these experiments, the imbibition within the internal pores could not be detected due to the dominant filling of the interparticle voids.

One might expect the imbibition in the third section to eventually reach a plateau with C_*pore*_ ≈ 0. However, no such behavior was observed, even after measurements lasting 6–10 h. This contrasts with previous findings reported in the literature.^34,62,63^ However, the experimental setup used in this study differs significantly from those previously reported. Typical rise times are on the order of hours to days.^34^ Consequently, detecting such a minute mass increase over extended periods is extremely challenging. Factors such as test tube movement, evaporation of the test liquid, and temperature fluctuations further complicate the long-term measurements. Another experimental limitation that could not be fully avoided is the adsorption of water from ambient air. This may reduce the effective pore radius and lower the surface energy of the CPG. Additionally, the imbibition of nonpolar liquids can be affected by unfavorable interactions with adsorbed water. The influence of vapor pressure on imbibition into porous materials and the adsorption from the surrounding vapor can only be neglected for nonvolatile liquids such as long-chain alcohols.^64^

#### Comparison of the Lucas–Washburn Coefficients

Examining C_*void*_ and C_*pore*_ presented in Table 4 reveals that C_*void*_ > *C*_*pore*_ for all measurements. For pCPG-OH, water imbibition yields the largest coefficients, followed by the aqueous C_10_E_4_ solution and *n*-octane. In contrast, the more viscous liquids, namely, the bicontinuous microemulsion and 1-octanol, exhibit the smallest coefficients, with no clear trend distinguishing which of the two possesses the greater LW coefficient. For pCPG-CH_3_, neither water nor the aqueous C_10_E_4_ solution shows measurable imbibition, yielding C_*void*_ = C_*pore*_ = 0. This observation deviates from the expected C_*LW*_ values. As discussed above, the surfactant concentration appears insufficient to form a complete monolayer on the pCPG-CH_3_ surface, which is an essential condition for the aqueous surfactant solution to penetrate the hydrophobic pores. The imbibition of *n*-octane is comparable for both hydrophilic and hydrophobic pCPGs. The LW coefficients for 1-octanol and the bicontinuous microemulsion, though smaller, remain within the same order of magnitude as those for the hydrophilic samples.

For the bicontinuous microemulsion imbibing into pCPG11 and pCPG50, the parameter C_*pore*_ is slightly larger in case of the hydrophilic surface. This difference disappeares in the case of pCPG100 where the mean pore size significantly exceeds the oil–water domain size of the bicontinuous microemulsion.

Overall, no significant differences are observed between the imbibition of the bicontinuous microemulsion and that of 1-octanol with its comparable viscosity and surface tension. This experimental observation was made within the limits of sensitivity and accuracy of the gravimetric measurement. Structural changes during the imbibition process do not necessarily manifest as changes in the rate of mass increase.

Although the macroscopic values are very similar, the microscopic origins of the viscosity differ. While in case of 1-octanol hydrogen bonding, van-der-Waals interactions of the alkyl-chains and transient short-range structuring of the molecules determine the viscosity, inside the microemulsion there is an additional structural contribution due to the surfactant interphase between the aqueous and the oil phase.

It is noteworthy that differences in the structure of the microemulsion in hydrophilic and hydrophobic pores were observed by means of small angle neutron scattering. This was measured in the equilibrated sample, but not during imbibition. The analysis of the SANS data indicated, that the structure remains more likely bicontinuous inside larger hydrophilic pores, while smaller pores may favor a partial phase separation. When the pores were hydrophobically modified, both scenarios, phase separation and maintaining the bicontinuous structure may be partially true.^43^

Since the theoretical coefficients C_*LW*_ were calculated for a comparison with C_*pore*_, they are considerably smaller than the experimentally obtained C_*void*_. While some deviations exist between C_*pore*_ and C_*LW*_, both remain within the same order of magnitude. This consistency supports the conclusion that the imbibition into the internal pore network is indeed observable in the third section of the spontaneous imbibition experiments.

Previous studies on the imbibition of alkanes and alcohols into nanoporous CPG structures^33,34^ demonstrated that the polar surface of hydrophilic pore walls promotes the formation of a permanent molecular layer, which in turn reduces the imbibition rate. These findings are supported by investigations on the surface melting of alkanes at planar interfaces.^65^ At planar hydrophilic surfaces the formation of a layered ordering inside the bicontinuous microemulsions.^10,66^ This structuring is supporting the flow along surfaces leading to the lubrication effect.^59,67,68^ The C_10_E_4_ molecules can adsorb at the hydrophilic and hydrophobic interfaces, effectively coupling the bicontinuous structure to the solid surface. This interfacial structuring contributes to an increased effective viscosity and, consequently, a pronounced slowdown of the imbibition process.

## Conclusions

The spontaneous imbibition of the microemulsion and its compounds into a packed column of pCPG has been investigated. Initially, a reproducible procedure was developed for the compaction of pCPG within the glass tubes utilizing a custom-designed 3D-printed device. This facilitated the achievement of a uniform packing of the pCPG. The imbibition of the test liquids was followed gravimetrically using the LW approach. The imbibition data show three distinct sections. Subsequent to the initial phase, the liquid ascends into the pCPG driven by capillary forces, following the characteristic LW $\sqrt{t}$ dependency. During the third phase, mass uptake continues to be observed, which also adheres to the $\sqrt{t}$ dependency but on a much slower time scale. The imbibition in the second section can be assigned to the liquid rise into the interparticle voids, while the imbibition observed in the third section corresponds to the rise inside the mesopores of the pCPG. The rate of imbibition is enhanced in smaller pores, consistent with the predictions of the LW equation. The surface polarity of the pCPG influences the imbibition behavior of water and aqueous surfactant solutions. However, the imbibition process of the bicontinuous microemulsion remains unaffected by the surface chemistry of the pCPG. In conclusion, the imbibition behavior of bicontinuous microemulsions can follow classical Lucas–Washburn imbibition behavior despite their complex internal nanostructure. The imbibition behavior of the bicontinuous microemulsion exhibits similarities to simple fluids with comparable macroscopic properties. These findings are important for applications of bicontinuous microemulsions in porous materials, such as in enhanced oil recovery. A fluid behavior, similar to that of a Newtonian fluid, renders the flow behavior more predictable.

## Supplementary Material



## Notes

Details of surface preparation and modification; physical properties of the used test liquids; surface energy determination (planar substrates); additional SEM imaging of the CPG particles; packing the CPG particle columns—method and results; results of additional Washburn measurements (PDF)
