# Supplementary material for: Spontaneous Imbibition of Bicontinuous Microemulsions into Hydrophilic and Hydrophobic Controlled Pore Glasses
Source: ACS Omega. 2026 May 20;11(21):31081–91. doi: 10.1021/acsomega.6c00570 (PMC13234799; doi:10.1021/acsomega.6c00570)
Supplement: Supplementary file 1 [file ao6c00570_si_001.pdf]

SUPPORTING INFORMATION: Spontaneous Imbibition of Bicontinuous Microemulsions into Hydrophilic and Hydrophobic Controlled Pore Glasses

Margarethe Dahl,† Larissa Doll,† Christian Tesch,† Benjamin Paul,† Jessica Hübner,† Brigitte Tiersch,‡ Thomas Hellweg,¶ and Stefan Wellert*,†

†*Technische Universität Berlin, Institut für Chemie, Straße des 17. Juni 135, 10623 Berlin, Germany*

‡*Universität Potsdam, Institut für Chemie, Karl-Liebknecht-Straße 24-25, 14476 Potsdam, Germany*

¶*Universität Bielefeld, Institut für Chemie, Physikalische und Biophysikalische Chemie, Universitätsstraße 25, 33615 Bielefeld, Germany*

E-mail: s.wellert@tu-berlin.de

Phone: +49 (0)30 31424958. Fax: +49 (0)30 31426602

Characterization of planar hydrophilic and hydrophobic surfaces

Surface preparation and modification

For laboratory experiments, like contact angle measurements, silicon wafer Si(100) with a thickness of (400±25) μm from MicroChemicals GmbH (Ulm, Germany) was cut into 20×20 mm pieces. For neutron reflectometry, circular silicone substrates with a diameter of 10 cm, thickness of 10 mm and a roughness < 1 nm were purchased from Nano Quartz Wafer GmbH (Langenzenn, Germany). The planar surfaces were cleaned with Ferasil solution (1 %) in an ultrasonic bath for 15 min. After drying under nitrogen stream, the substrates were immersed in Piranha solution (v/v = 1:1; H_2SO_4, H_2O_2 $_{aq.}$) for 20 min to achieve an oxide layer. The hydrophilic surfaces (Si-OH) were obtained after rinsing with water and drying under nitrogen stream.

The silicone substrates were dried in an oven at 150 °C for 1 h to remove water residues. Anhydrous toluene (60 mL) was degassed by purging it with nitrogen for 30 min while stirring. Dichlorodimethylsilane (DCDMS) (0.72 mL) was added while stirring for 10 min. The silicone substrates were immersed in the toluene solution for 1 h at room temperature. Any DCDMS residues were removed by placing the substrates in an ultrasonic bath for 10 min in toluene. After cleaning the substrates with acetone and water in the ultrasonic bath for 10 min each, the hydrophobic planar surfaces Si-CH_3 were obtained.

Physical properties of the used test liquids

Table S1: Probe liquids and its total free energy σ_s, polar σ_s^p and dispersive component σ_s^d taken from[1,2]

probe liquid	σ_s (mN/m)	σ_s^p (mN/m)	σ_s^d (mN/m)
water	72.8	51.0	21.8
glycerol	62.7	41.5	21.2
diiodomethane	50.8	0.4	50.4

Surface energy determination

The contact angle on hydrophilic and hydrophobic solid surfaces of the microemulsion and its components was studied using the sessile drop method with an OCA15+ (Optical Contact Angle) from DataPhysics Instrument equipped with a temperature-controlled sample chamber and syringe dosing unit. The drop volume of the test liquids was set to a few μL depending on the liquid and the contact angles were analyzed with the software SCA20 from DataPhysics. The equilibrium contact angle θ is the angle that is formed at the contact line between the solid, liquid, and vapor. The relation with the surface energy of the surface σ_s, the surface tension of the liquid σ_l, and the solid-liquid interfacial energy σ_{sl} is described by the Young equation:

$$\sigma_l \cos\theta = \sigma_s - \sigma_{sl} \quad . \tag{1}$$

Dynamic contact angle measurements were performed to determine the advancing contact angle θ_a,the receding contact angle θ_r and the contact angle hysteresis by inflating and deflating a sessile drop of the test liquid with a needle using the dosing unit of the instrument. The volume was varied by $\pm$ 2 μL. The drop profile was analyzed with the ellipse model or the tangent leaning method for contact angles below 30 °. The measurement was repeated three to five times, and the mean value was calculated. The standard deviation was then used as the uncertainty of the contact angle.

The surface modification of the planar solid substrates was verified by measuring the static contact angle of different probe liquids. According to the OWRK method, the surface free energy can be split into its polar and dispersive parts, as expressed in Eq. 2.[3–5] Contact angle measurements with probe liquids with known dispersive and polar components allow the determination of the surface free energy. The mean contact angle was calculated based on the analysis of three to five droplets per surface. The standard deviation was used as the uncertainty measure. After inserting Eq. 2 in Eq. 1 and linearization, σ_s^d and σ_s^p can be obtained from the fitted slope and intercept. The probe liquids and their total free energy

with the polar and dispersive components are listed in Table ??.

$$\sigma_{sl} = \sigma_s + \sigma_l - 2\left(\left(\sigma_s^d \sigma_l^d\right)^{1/2} + \left(\sigma_s^p \sigma_l^p\right)^{1/2}\right) \tag{2}$$

Inserting equation 2 into equation 1 yields the so-called OWRK equation:

$$\frac{\sigma_{sl(1+cos(\Theta))}}{2\sqrt{\sigma_l^p}} = \sqrt{\sigma_s^d} + \sqrt{\sigma_s^p}\sqrt{\frac{\sigma_l^p}{\sigma_l^d}}. \tag{3}$$

Plotting equation S3 according to y=mx+n yields n=$\sqrt{\sigma_s^d}$ and m=$\sqrt{\sigma_s^p}$.

Table S2: Experimentally determined surface free energy σ_s with its polar σ_s^p and dispersive σ_s^d components of hydrophilic (Si-OH) and hydrophobic (Si-CH_3) planar surfaces

surface	σ_s (mN/m)	σ_s^p (mN/m)	σ_s^d (mN/m)
Si-OH	73.9 ± 0.2	37.6 ± 0.1	36.2 ±0.1
Si-CH_3	24.7 ± 0.2	1.2 ± 0.1	23.7 ± 0.1

SEM imaging of the CPG-particles

Experimental details

Scanning electron microscopy

SEM images of the pCPG were recorded on a JEOL JSM 7401F with a magnification of 10 000 and 50 000 using a lower secondary electron detector operated at an accelerating voltage of 5 keV. The measurements were conducted by Benjamin Paul (Technische Universität Berlin).

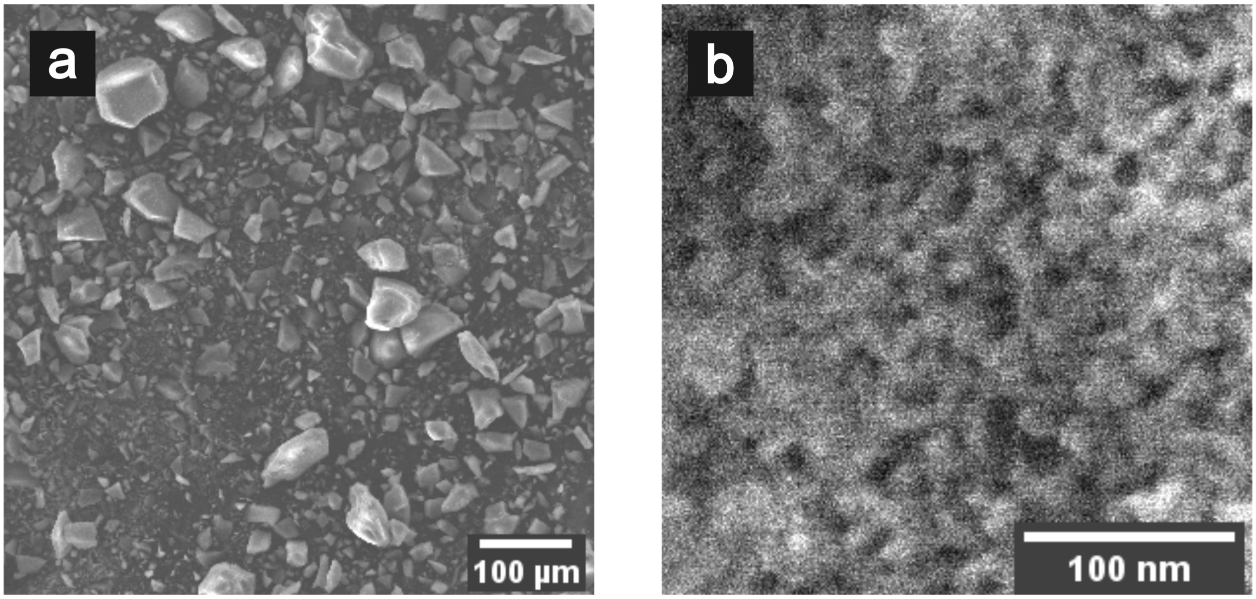

Figure S1: SEM micrographs of hydrophilic pCPG11 at a magnification of a) 50 and b) 50k.

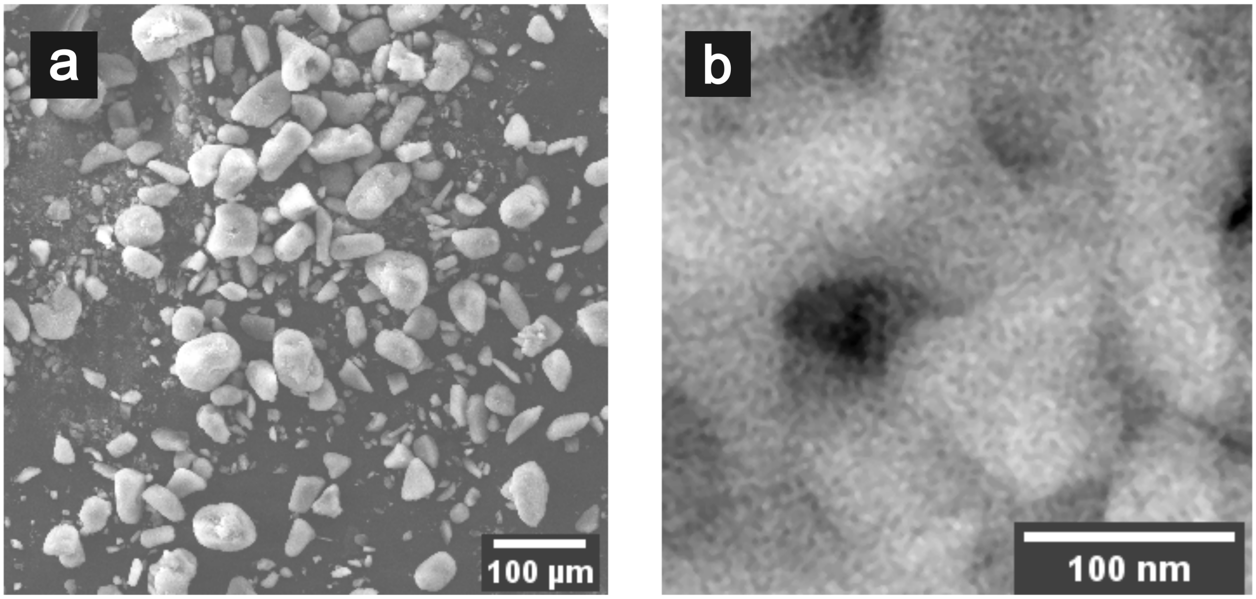

Figure S2: SEM micrographs of hydrophilic pCPG100 at a magnification of a) 50 and b) 50k.

Results

Packing the CPG particle columns

A commonly reported limitation of the Lucas–Washburn (LW) approach is the inconsistent packing of porous powders.[6] To ensure reproducible imbibition measurements, it is essential

to compact the particles in a uniform and robust manner. Beside other approaches, a standard method for compacting powders involves tapping the filled glass tubes on a hard surface.[7] However, manual tapping is not only time-consuming but also prone to inconsistencies, such as variations in tapping height or accidental dropping of the tubes. To address these issues, a custom designed, 3D-printed tapping device was developed to automate the tapping process and ensure consistent and homogeneous packing of the powder column. A schematic of the device is shown in Figure S3. The glass tubes are securely held in a sled (yellow in Figure S3) specifically designed to match their dimensions. The sled is lifted and dropped by a rotating cam disk (purple) driven by a DC motor. Both the number of taps and the rotation speed are controlled via a Python program. For consistent compaction, each filled tube was subjected to 150 tapping cycles from a height of 2 cm. The sled is easily removable and can be replaced

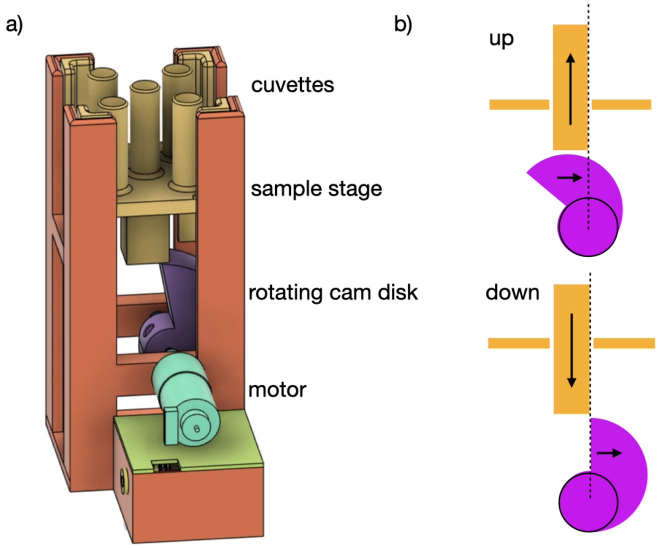

Figure S3: a) Schematic presentation of the 3D-printed device for tapping the powder filled glass tubes. b) Tapping mechanism using a rotating cam disk to lift and drop the cuvettes.

to accommodate containers of different sizes and shapes, making the system flexible and adaptable for various applications.[8] Overall, the device offers a reliable and reproducible solution for compacting powders in experimental setups. To determine the optimal number of taps required for a reproducible packing procedure in the imbibition studies, the filling level of different pCPG powders was measured as a function of the number of taps. The

results for pCPG50-OH and pCPG100-OH are shown in Figure S4. A significant decrease in the filling level was observed within the first 20 taps. After approximately 50 taps, the filling height stabilized and showed no further reduction with additional tapping. To ensure consistent and thorough compaction across all experiments, 150 tapping cycles were applied to each filled tube. Although the initial filling differs, which might be influenced by the shape and size distribution of the grains, already after 50 cycles the minimal column heights were achieved by compacting the grains.

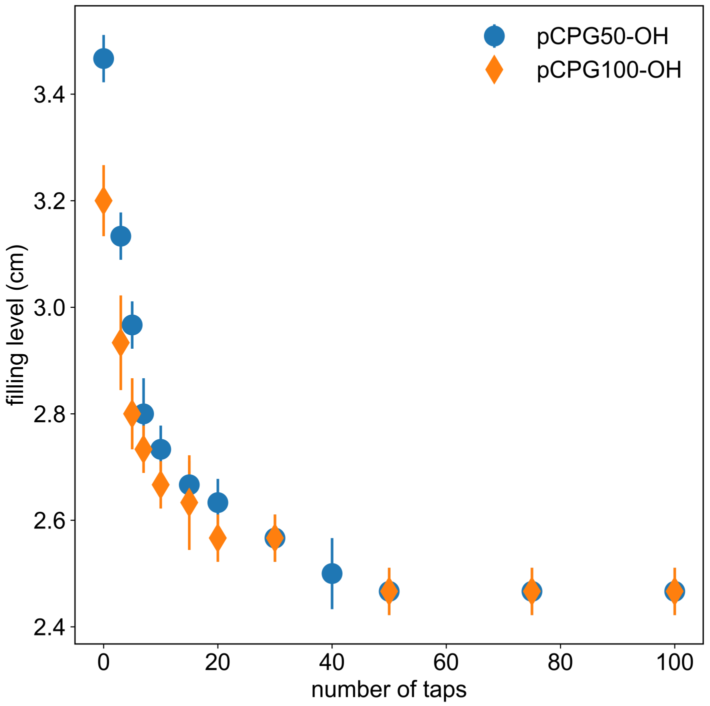

Figure S4: Filling level of pCPG50-OH and pCPG100-OH as a function of the number of taps after the first 100 cycles.

Results of additional Washburn measurements

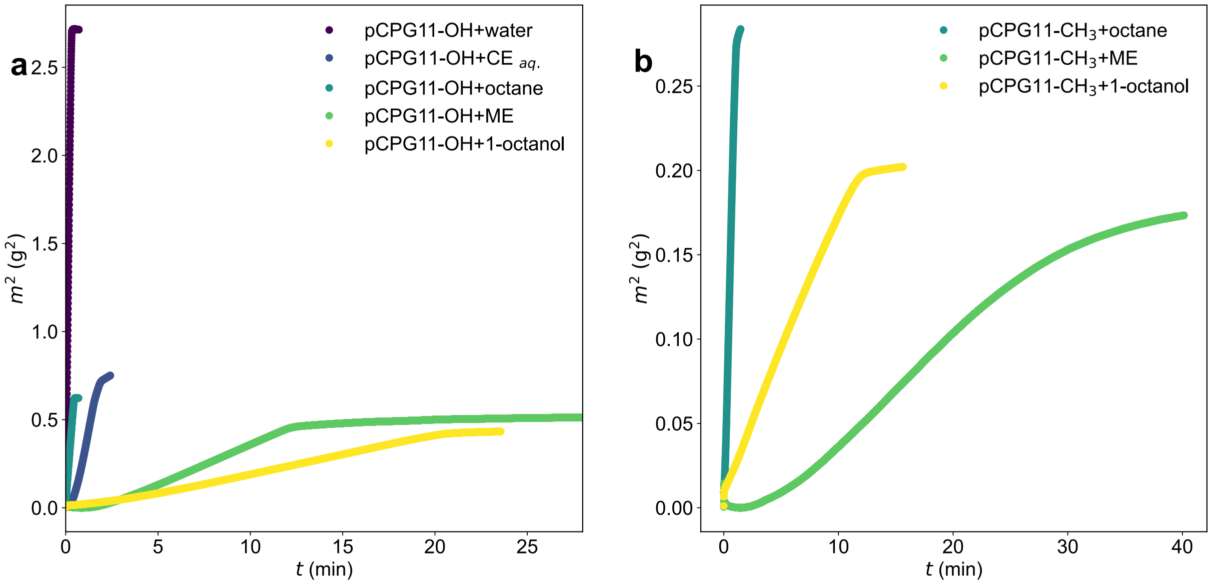

Figure S5: Imbibition of $C_{10}E_4$/water/n-octane, its component and 1-octanol into pCPG11-OH (a) and pCPG11-CH_3. Water and aqueous $C_{10}E_4$ solution do not imbibe into pCPG-CH_3 and are therefore not shown.

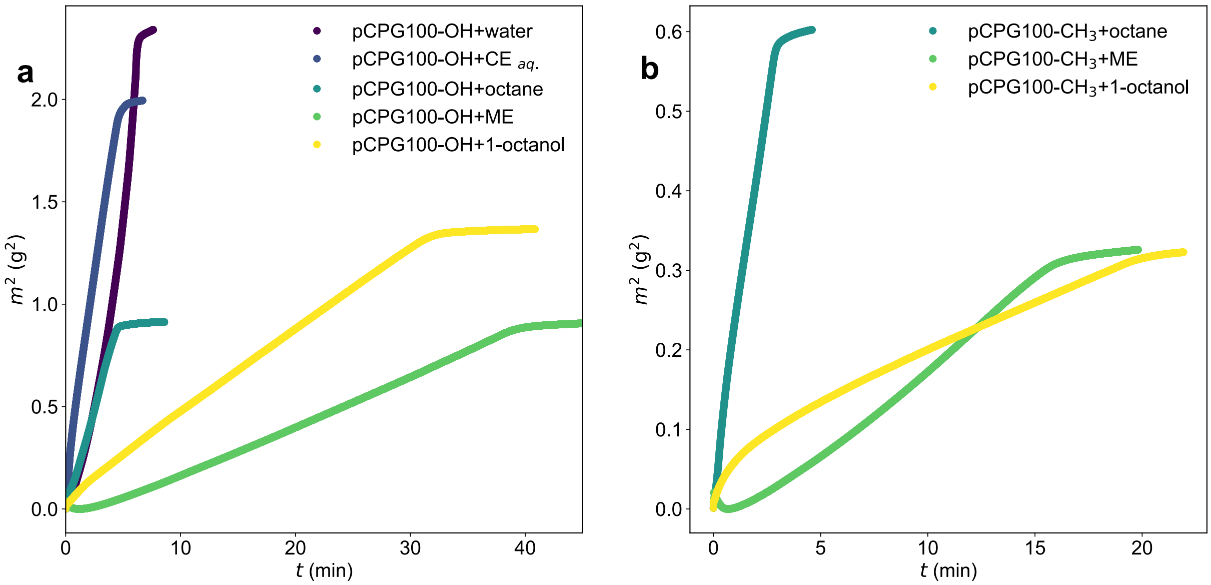

Figure S6: Imbibition of $C_{10}E_4$/water/n-octane, its component and 1-octanol into pCPG100-OH (a) and pCPG100-CH_3. Water and aqueous $C_{10}E_4$ solution do not imbibe into pCPG-CH_3 and are therefore not shown.

Literatur

(1) Busscher, H.; Van Pelt, A.; De Boer, P.; De Jong, H.; Arends, J. The effect of surface roughening of polymers on measured contact angles of liquids. *Colloids and Surfaces* **1984**, *9*, 319–331.

(2) Jańczuk, B.; Białlopiotrowicz, T. Surface free-energy components of liquids and low energy solids and contact angles. *Journal of Colloid and Interface Science* **1989**, *127*, 189–204.

(3) Owens, D. K.; Wendt, R. C. Estimation of the surface free energy of polymers. *Journal of Applied Polymer Science* **1969**, *13*, 1741–1747.

(4) Kaelble, D. H. Dispersion-Polar Surface Tension Properties of Organic Solids. *The Journal of Adhesion* **1970**, *2*, 66–81.

(5) W. Rabel Einige Aspekte der Benetzungstheorie und ihre Anwendung auf die Untersuchung und Veränderung der Oberflächeneigenschaften von Polymeren. *Farbe und Lack* **1970**, *77*, 10.

(6) Alghunaim, A.; Kirdponpattara, S.; Newby, B.-m. Z. Techniques for determining contact angle and wettability of powders. *Powder Technology* **2016**, *287*, 201–215.

(7) Kirdponpattara, S.; Phisalaphong, M.; Newby, B.-m. Z. Applicability of Washburn capillary rise for determining contact angles of powders/porous materials. *Journal of Colloid and Interface Science* **2013**, *397*, 169–176.

(8) Tesch, C. Tapping Device. `https://github.com/tesch-ch/tapping_device`.
